# Interviewing the Internalized Other and the Distributed Self

**DOI:** 10.1111/famp.70025

**Published:** 2025-03-18

**Authors:** Karl Tomm

**Affiliations:** ^1^ University of Calgary and Calgary Family Therapy Centre Calgary Alberta Canada

**Keywords:** distributed self, internalized other, reflexive questions, social constructionism

## Abstract

A unique psychotherapeutic method of Internalized Other Interviewing (IOI) has emerged over the last 35 years. It is based on a social constructionist and bringforthist perspective in which a *person* as a self‐aware individual is seen to arise through a rich history of interaction with other human beings. If a therapist can apply this understanding and conceive of the *self* as constituted by an internalized community in one's memory, it becomes coherent to interview any member of that inner community as an *internalized other* within the client. As a result, the possibilities for intervention in a client's relationships are expanded. If the IOI method is used in the presence of the *actual other* in conjoint work, the latter could meet their *distributed self* as they exist in the interviewee, and even more change possibilities arise. This paper focuses on applying the method in clinical practice using examples, transcripts, and commentary.

## 1 Introduction

*Internalized Other Interviewing* (IOI) involves asking questions to an interviewee who embodies and speaks from the internalized experience of another person. While this method of interviewing may be employed in a variety of contexts, such as coaching, supervision, and training, this paper will focus on its application in systemic therapy. The therapist addresses the embodied *internalized other* (IO) within the client to bring forth selective aspects of the IO's experiences to enable relational healing and wellness. The IO is usually someone the client knows well, such as a family member, a friend, a colleague, or a workmate. Thus, the interviewee usually has a significant history to draw upon when responding in the voice of the internalized other.

Let us take for example, George^1^, who is referred for individual therapy because of yelling and screaming when trying to parent his teenage daughter, Bonnie. He realizes that his angry outbursts are not appropriate, but he can't help himself when he experiences her as so disrespectful and rebellious. The more the father pressures the daughter to comply the more she resists, and the more she resists the more he tries to force her to comply. Thus, one focus in the therapy could be to deconstruct the pathologizing interaction pattern (PIP; Tomm et al. 2014) of the father demanding obedience coupled with the daughter resisting and rebelling. To help break this cycle George might be invited to speak from the *I position* of Bonnie who exists as an IO within George's memory and imagination. The therapist encourages George to respond to IO questions from his intuitive hunches regarding Bonnie's inner experiences: “Bonnie, what does it feel like inside when your dad raises his voice and insists that you listen to him? … And when you feel that way, are you more, or less, likely to do what he says?” The more honest George is in his intuitive answers as Bonnie, the more likely her probable feelings of fear, anger, and/or outrage will become available to him internally to help him ameliorate his habit of yelling and screaming. Questions addressed to the IO constitute a stronger invitation towards empathic understanding than interpersonal perception questions to George like “How do you think she feels about your yelling?” which may also be helpful but tend to remain more intellectual.

Another focus in the IOI could be to co‐construct more wellness in the relationship. The therapist might ask the IO: “Bonnie, what could your dad do differently that might make you feel more willing to cooperate? … What else could he try, Bonnie?” During the interview the therapist uses Bonnie's name repeatedly to address his IO when speaking with George, to help him remain grounded in his experience of her experience. IOI is not conceived as a role play where George's responses might manifest the surface of Bonnie's behavior (such as parroting her words). The intent is to bring forth Bonnie's inner experience, from as deeply as George is able to go. In responding to these questions, George draws upon past and anticipated interactions between himself and Bonnie, stories he has heard about her, and his overall impressions about Bonnie's thoughts, feelings, intentions, hopes, etc. The specific questions that the therapist asks may influence George's understanding of Bonnie, his understanding of himself from her perspective, and his understanding of his own understanding of their relationship.

The process of interviewing the internalized Bonnie within George could also be enacted while Bonnie is physically present to observe. If she was to witness such an interview, she would be afforded the opportunity to meet a version of her *distributed self* (DS) – namely, how she exists in her father—and perhaps hear her DS saying things like: “I can't stand it when he gets so controlling! … It makes me furious!” Such witnessing usually turns out to be a fascinating experience for the observing person and could have a variety of effects on her. Our identities are in part constructed by how others reflect onto us who we are. Versions of ourselves are distributed within all those who know us (as well as within all those who know of us by reputation). At the very least, hearing George respond in Bonnie's *voice* gives the observing Bonnie an opportunity to reflect upon and contrast her own reactions to the therapist's questions and to notice when George's answers‐as‐Bonnie fit, or do not fit, for her. Furthermore, the therapist's questions addressed to the internalized Bonnie‐in‐George may trigger changes directly within the observing Bonnie (who is not actively contributing to the conversation in the moment). Indeed, a therapist might deliberately ask certain questions to the internalized other where the intent is to embed a suggestion for the observing actual other: “So Bonnie, suppose you were to tell your dad afterwards how terrified you felt when he shouted at you, can you imagine that he might think twice next time, about how he talks to you?” Some of the nuances involved in interviewing the DS will be explored later. Suffice it to say here, every internalized other is someone else's distributed self, and the IOI process is significantly more complex when George and Bonnie are both physically present.

The judicious use of this unusual method of interviewing can have surprisingly beneficial effects, especially when a therapy has come up against an impasse. What is extremely important, however, are the specific questions that are asked of the IO. They can make an enormous difference to the impact on both George and Bonnie, as well as on their relationship. Reflexive questions (Tomm 1988) are always given priority in this process with the intent to co‐construct more individual and relational wellness. It is beyond the scope of this paper to address the process of reflexive questioning, which has been described elsewhere (Tomm 1987). However, the examples in the transcripts that follow will illustrate the use of IO questions in the flow of an IOI conversation.

I have been conducting internalized other interviews from time to time for 35 years and have taught the method in the international family therapy community throughout that time, but I have not written much about it. Several colleagues have taken the initiative to publish papers on the method (Burnham 2000; Emmerson‐Whyte 2010; Epston 1993; Haydon‐Laurelut and Wilson 2011; Hurley 2006; Lysack 2002; Moules 2011; Mudry et al. 2015; Nyland and Corsiglia 1993; Paré 2001) and at least three theses have been written about it (Akdeniz 2021; Brosh 2007; Houston 2008). They cover the topic well, and I felt little need to write more about it. However, I have been strongly encouraged to document my contributions in developing the method and to share examples of my own application of the approach. So, I decided to use the opportunity in my final sabbatical to describe my occasional use of IOI.

## 2 Origins of the IOI Method

It is often not entirely clear how one comes to a particular idea or clinical practice. In retrospect, I suspect the seeds of my work in IOI began germinating after some contact with Fritz Perls in 1968. Perls founded Gestalt Therapy (Perls et al. 1951), and I was fascinated when I heard him speak about the empty chair technique during my first year of psychiatry residency.^2^ In Gestalt work, the therapist asks the client to speak from the experience of *the other* when they move to sit in the empty chair. While there are similarities between IOI and the Gestalt two‐chair technique, there are differences in both theory and practice. For one thing, Gestalt work arises out of intrapsychic psychoanalytic assumptions, whereas IOI work arises out of interpersonal systemic assumptions. In practice, the other in Gestalt work is always projected into the empty chair, while in IOI work the other always remains within the person of the self.

There were many additional influences along my path towards interpersonal, systems‐oriented IOI. I have shared details elsewhere about my professional journey from traditional psychiatry to family therapy and on to systemic therapy (Bubenzer et al. 1997; Collins and Tomm 2009). Briefly, it was the influence of Nathan Epstein and the McMaster group (Epstein et al. 1978) that first enabled me to shift my focus in psychiatry from working with individual clients to working with families as systems. Then the Milan team in the late 1970s (Selvini et al. 1978) and an exploration of Gregory Bateson's theory of Mind (Bateson 1972) ushered me into systemic work. Michael White's narrative practices (White and Epston 1990, White release 2007) and Humberto Maturana's theory of knowledge (Maturana and Varela 1979, 1987) took me further into systemic understandings in the early 1980s. The terms *system* and *systemic* are used in various ways in the family therapy literature. I use system to describe observed systems where the observer is separate from the observation, and systemic to refer to observing systems where the observer is part of the system being described. As part of the therapy system, I see myself as systemic in formulating relevant reflexive questions.

Ultimately, it was a story shared by David Epston that finally prompted me to begin experimenting with IOI as such. On a visit to the Family Therapy Program in Calgary in 1988, David recounted an interview with a highly conflicted couple where he had used “cross‐referential questioning” (Epston 1993, 185) to speak to the wife as if she were the husband and vice versa. I subsequently tried to use David's cross‐referential questions in my clinical work, but they didn't seem to work for me. A year later, after a conference I organized in Calgary to compare the interviewing styles of Michael White and Gianfranco Cecchin, it occurred to me that if a therapist construed the psychological self of a client as constituted by memories of past social interactions to maintain a relatively stable internal community, it became coherent to interview any member of that community within the client. By re‐describing these questions as *internalized other questions*, I was able to formulate and ask them more easily. Giving a good label to a certain kind of question is like attaching a good handle to a tool—it becomes easier to use. I shared my enhanced experience with David, who agreed to the re‐labeling, and we presented a workshop together on IOI at the first Therapeutic Conversations Conference in Tulsa, Oklahoma, in 1990 (Epston 1993).

### 2.1 Clinical Vignette #1—IOI of a Mother Within a Middle‐Aged Man

Some years ago, I employed the method of IOI in a demonstration interview at a workshop I conducted for colleagues. A workshop participant had asked one of her clients, Bob, a white, middle‐aged, cisgender, single male, if he would accept being interviewed live in front of a group of professionals. Bob readily agreed since he felt good about his progress in therapy. He had a long history of behavioral difficulties from childhood through his adult years. During his early adolescence, his mother had taken him to a clinic for assessment where he was diagnosed with ADHD and placed on Ritalin. This turned out to be the beginning of a chemical lifestyle for Bob. Over the years, he experimented with a variety of drugs and eventually became a heavy drinker. However, in his middle years, he joined AA and was able to get into an ongoing support program as well as long‐term individual therapy. He gradually weaned himself off all drugs and alcohol, and for the first time, was able to maintain a steady full‐time job. He was especially pleased that he had managed to remain clean for several months.

It just so happened that a few days prior to my interview he developed a head cold which went into his chest. This resulted in him coughing at night, so he had difficulty sleeping. The day before the interview he called his physician and asked for some cough syrup. In the call he specified that he wanted codeine in the cough syrup; codeine is an opiate known to be a cough suppressant. The doctor complied and prescribed the medication as requested. That night, before the interview, he took some cough syrup, and then some more, and then more, etc. Suddenly he realized that he was taking the cough syrup for the codeine kick and not just for the cough! This realization upset him terribly—he had struggled for so many years to overcome his addictions. He became extremely despondent and began contemplating suicide. Fortunately, he had the presence of mind to phone his AA sponsor who cautioned him: “Don't be rash, just sleep on it, and we can talk about it tomorrow.” Bob took this advice, and the next morning remembered the commitment to attend the workshop to be interviewed, where this story emerged.^3^

No one had criticized or judged Bob for taking the cough syrup for the codeine kick. Indeed, no one even knew he had done so. Yet he felt so heavily judged that he wanted to kill himself. I speculated that an internal pattern of intense self‐criticism had provoked his suicidal thoughts. This self‐judgment seemed profoundly unfair, yet I had to be careful because I did not want to criticize Bob for being so self‐critical. Instead, I imagined some relational origins of the criticism and anticipated that Bob might afford me the opportunity to explore those origins, begin deconstructing them, and perhaps co‐construct some relational healing to displace the criticism.

#### 2.1.1 Interview

Karl “Among all the people that have known you, who do you imagine would have been most upset if they knew you had taken the cough syrup for the codeine kick?” With this question I invited Bob to help me identify a significant person in his life who may be relevant to the current dilemma.

Bob (After a long pause) “Probably my mother.” Bob went on to explain that he had a very difficult relationship with his mother. She now lived in another city, and they were no longer on speaking terms.

Karl “What's your mother's first name?” It is essential to use the name of the IO to help the client become grounded in the experience of the other.

Bob “Agnes”

Karl “Could I speak with Agnes for a while?” Bob conveyed some confusion, and I went on to explain the process of IOI.

Bob “Sure, I'll give it a try.”

Karl Now addressing Agnes within Bob, “Agnes, what was Bob like as a child when you were raising him?” My hope here was for Bob to give me some relevant history out of which his self‐criticism may have developed.

Bob Answering as Agnes, “Oh, he was a real troublemaker! … Getting into trouble all the time. … Always raising havoc!”

Karl To Agnes within Bob, “How did you respond to him when all this was going on, Agnes?” This behavioral effect question served to disclose a problematic interaction pattern between the mother and son.

**FIGURE 1:**
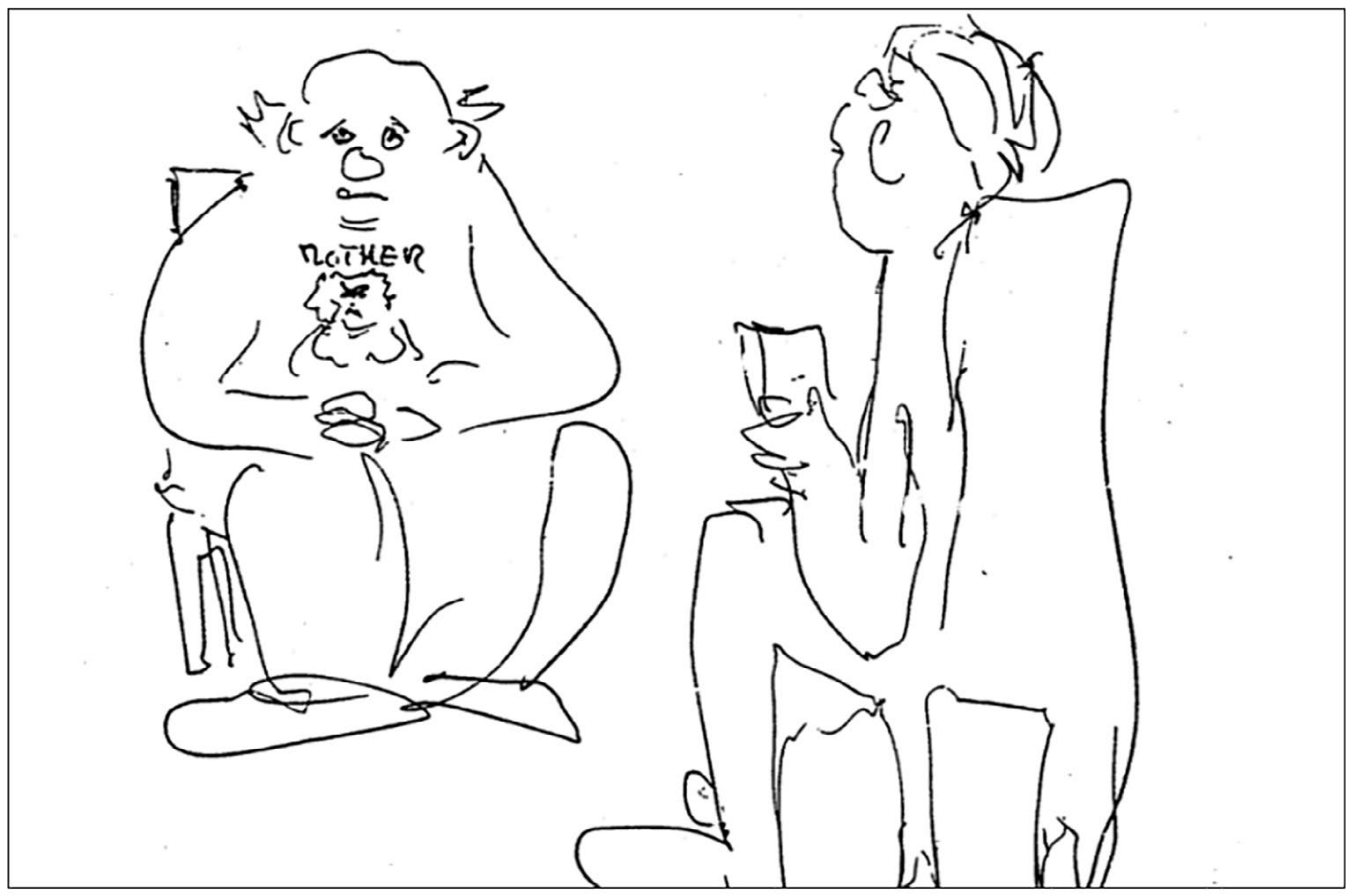
Interviewing the internalized mother.

Bob: Answering as Agnes, “I tried to correct him … I told him again and again that what he was doing was wrong … that it was bad … that he needed to be punished.” The internalized mother within the client in Figure 1 was drawn by a Swedish colleague who heard me recount this story. It illustrates how visualizing the IO inside Bob can help the therapist remain focused on addressing the IO.

Karl To Agnes within Bob, “Agnes, would you say then, that you had to rely on criticism a fair bit as a way to raise him?” This question introduced the distinction of a process of criticism but located it in an interaction pattern outside Bob.

Bob Answering as Agnes, “For sure. … But then he'd complain that I was being unfair, and he'd act out even more”

Karl To Agnes within Bob, “Agnes, suppose you came to realize that Bob may have internalized the pattern of criticism as a way to deal with problematic behavior, and that he had come to a place now where he was criticizing himself so strongly, for taking the codeine last night, that he was contemplating his own suicide, would you have any regrets in having depended so much on criticism to raise him?” This question introduced a hypothesis about the relational origins of the criticism habit and pointed towards a possible corrective initiative.

Bob Answering as Agnes after a long pause, “Well … maybe.”

Karl To Agnes within Bob, “Say you did express regret, and possibly even apologized to Bob for relying so much on criticism as a way to raise him, do you imagine he could ever forgive you?” This embedded suggestion question invited Bob to consider embracing a powerful healing pattern of *apologizing coupled with forgiving*. Tears welled up in Bob and he began to weep. Karl gave space for Bob to process his emotion. Eventually, Bob spoke as himself, spontaneously.

Bob Answering

“Yes, I think I could forgive her.”

Karl To Bob as Bob, “Do you think it might be fair to forgive yourself as well?”

Bob “Yes. … I guess so.”

#### 2.1.2 Comments Regarding Vignette #1

Bob's tears reflected a significant shift in the meaning‐making within his internalized community, especially in his relationship with his internalized mother and in his relationship with himself. It is not possible to specify exactly what changed in his nervous system. However, there was a clear difference in his attitude towards himself, with more self‐acceptance. Most importantly, his suicidal impulses evaporated. My accepting attitude towards him undoubtedly was helpful, but my hunch is that the series of questions were much more influential in perturbing (in a good way) his deeply embedded memories of past interactions with his mother. A client's memories are always implicitly available as immanent affordances to be drawn upon to create opportunities for therapeutic initiatives (Tomm and Taylor 2025). What is noteworthy in this interview is my confidence that Bob already possessed sufficient life experiences to bring forth the relational healing pattern of *apologizing coupled with forgiving* to displace the pathologizing pattern of *criticizing coupled with misbehaving* (Tomm et al. 2014). When a therapist has confidence that a client already has the resources they require for healing, he or she will search for them and, of course, is then much more likely to find them and bring them forth to be applied in the current situation.

### 2.2 Clinical Vignette #2—IOI of a Teenaged Daughter Within a Mother

I had been invited to consult in a family therapy session after an episode of violence when the elder of two adolescent daughters (Alice) assaulted the younger sister (Brenda), who sustained injuries that required a visit to the hospital. The parents had separated when the girls were quite young. Intense sibling rivalry developed between the girls as the mother raised them (mostly on her own). Both girls and their stepfather (a recent addition to the family) declined the invitation to attend the consultation. Only the mother, Ethyl, came to the interview. The following transcript was taken from a video recording of the meeting and edited for readability.^4^

#### 2.2.1 Interview

Ethyl Early in the interview, Ethyl asked me

“What do I do with her (Alice) if she becomes violent again?”

Karl “It might be helpful to discuss this with her in advance when she's not upset. … If she's not able to exercise inner control, it might be reasonable to agree to call the police to provide some outer control … that's what the police are there for.”

Ethyl “But what can I do to calm her down when she gets upset?”

Karl “That's a good question. … It depends on what's going on for her at the moment.”

Ethyl “I don't understand what's going on for her. She thinks she's right all the time. No one is right all the time!” It was at this point that it occurred to me that some IOI might be helpful. I speculated that if the mother could empathize better with her daughter's experiences, she might be able to make better decisions in the moment about how to respond when Alice gets upset.

Karl “Are you open to an experiment? Could I speak with Alice as an internalized other within you, to try to help you get more in touch with what might be going on for her?” Introducing the method as an experiment, rather than as something that will help, avoids activating unrealistic expectations.

Ethyl Answering hesitantly, “OK”

Karl “Alright, let me speak with you as if you were Alice. I'm not asking you to role play her outer behaviour, but to speak from your experience of her inner experience, as deep as you're able to go.”

Karl Now addressing the daughter by name within Ethyl, “Alice, how do you feel about us bringing you into this situation with your mom?” If Alice were present in the interview, I would not use this opening question, assuming she would probably protest if she didn't want me to proceed. With a client alone, however, I usually begin with this question to probe for possible constraints to the process.

Ethyl Answering slowly as Alice, *“*I don't want you to do it.” This response is not typical and calls for some exploration. Usually, the internalized other readily accepts the experiment.

Karl To Alice within Ethyl, “Alice, why wouldn't you want me to do this … to help your mother understand you better?”

Ethyl Answering as Alice, “Everyone is against me …”

Karl To Alice within Ethyl, “But Alice, in talking with your mom earlier I got the impression that she wants to have a better relationship with you.” A comment to suggest common interests.

Ethyl Answering as Alice, “She just says that; she likes Brenda more than she likes me.” The rivalry between the sisters becomes apparent.

Karl To Alice within Ethyl, “Alice, can you believe that I would want you and your mom to have a good relationship?” A closed question attempting to bring forth some common ground.

Ethyl Hesitating and then answering as Alice, “… OK.” I took this as permission to proceed.

Karl To Alice within Ethyl, “Alice, when you and your sister were growing up with your mom, did you have the impression that your mom tried to treat the two of you the same?” This question came from my prior clinical experience of a common pathologizing pattern in families where there is intense rivalry between siblings. Parents sometimes unwittingly feed sibling competitiveness by trying to treat their children the same, which has the inadvertent effect of stimulating the children to make finer discriminations of differences and consequently complaining more about favoritism, triggering the parents to try harder to treat them the same, etc.

Ethyl Answering as Alice, “She tried to treat us the same, but I was closer to her.” My hunch is confirmed, and the competitiveness is still evident.

Karl To Alice within Ethyl, “Alice, would you prefer that she try to treat you the same or would you prefer that she treat you and your sister as unique and different?” This bifurcation question reflects an attempt to potentially bring forth a possible healing pattern of accepting differences in the mother‐daughter relationships to displace the competitiveness.

Ethyl Answering as Alice, “Unique and different.”

Karl To Alice within Ethyl, “What is unique and special about your relationship with your mom?” This question is intended to thicken and stabilize the now preferred pattern of accepting differences in how the mother treats each of the two daughters.

Ethyl Answering slowly as Alice, “I'm not sure.”

Karl To Alice within Ethyl, “Alice, what is it that your mom likes about you as a person … that she feels warmly about, when she thinks of you?” This question reflects an attempt to help Alice‐within‐Ethyl articulate a difference that would be acceptable. However, it also invites Ethyl to disclose her intuition about how she exists within her daughter. In other words, it enables Ethyl to begin meeting some aspects of her distributed self (more on this later).

Ethyl Answering as Alice, “I don't think she does.”

Karl To Alice within Ethyl, “Really? … What do you like about your mom? What do you appreciate about her? … What else? … What holds you back from showing this appreciation to your mom, Alice?” These questions are intended to bring forth and foreground positive aspects of the relationship between Alice and Ethyl. They usually have strong affirming effects on the interviewee. Indeed, Ethyl was moved to tears at this point, so I pause before moving on.

Karl To Alice within Ethyl, “Alice, your mother mentioned earlier that it's important for you to be right. What kind of relationship do you have with mistakes? Are you able to learn from mistakes?” These questions invite Ethyl to enter more deeply into Alice's need for being right.

Ethyl Answering as Alice, “When I make a mistake, I get upset. I get angry.”

Karl To Alice within Ethyl, “Can you imagine that if you could learn from a mistake, that you could feel good about the learning?”

Ethyl Answering as Alice, “I don't recall learning … I just end up blaming myself … I get depressed.”

Karl To Alice within Ethyl, “Where does that self‐blame come from Alice? Did you invent it, or did someone say something to you about it?”

Ethyl Ethyl encounters difficulty imagining Alice's experience here and breaks out of the internalized other interviewing process to speak as herself

“I … ah … I don't have any insight about her. I don't know how she thinks.” I have often noticed that when a client struggles to formulate a response in the voice of the other and spontaneously breaks into their own voice instead, the specific content issues at those points are usually highly relevant to the situation and often benefit from further exploration.

Karl To Ethyl as Ethyl, “I realize this is extremely difficult. … Remember that the Alice within you belongs to you. Try not to get stuck on what the outer Alice might think and feel. Allow your inner Alice to answer freely to explore her future possibilities. … Are you willing to work on this a little further?” I assume that if Ethyl allows her internalized Alice to evolve towards a more mature position, chances are that in their future interaction Ethyl might invite the actual Alice into that maturity.

Ethyl Answering as herself, “OK.”

Karl To Alice within Ethyl, “Alice, would you like to become the kind of person who could learn from her mistakes? … How do you imagine you could you do so?”

The interview continues exploring the issue of rightness a bit further and then shifts to focus on what the mother could do differently from the perspective of the internalized Alice “If your mom could make some changes in the way in which she relates to you, what change would you appreciate the most, Alice?” This is an extremely useful question to ask the internalized other since it helps to clarify what constructive initiatives Ethyl could take in her future interaction with Alice that probably would be meaningful for their relationship.

Ethyl Answering as Alice, “I'd like my mom to listen to me more.”

Karl To Alice within Ethyl, “And what would that look like, Alice, if that were to happen?” This follow‐through question helps Ethyl operationalize Alice's preferences in specific behaviors.

Ethyl Answering as Alice, “She could agree with me more.”

Karl To Alice within Ethyl, "What other changes would you appreciate if your mother were willing and able to make further changes in how she release to you?" The IOI continues for several minutes with Ethyl spontaneously breaking out of the IO from time to time. To Alice within Ethyl, “What other changes would you appreciate if your mom was willing and able to make further changes in how she relates to you?” This is simply an invitation to expand on the possibilities for constructive initiatives that Ethyl could consider. The IOI interview continued for several more minutes to further explore the themes of competitiveness, rightness, and agreement, with Ethyl spontaneously breaking out of the IOI from time to time.

Karl Ending the IOI, “Thank you Alice for responding so thoughtfully to my questions. … Hello again Ethyl. … What was it like for you to speak from Alice's experience?”

Ethyl Answering as herself, “That was hard! I really don't know what's going on inside her.”

Karl To Ethyl as Ethyl, “What's your best guess about what percent of your answers as Alice you imagine she would agree with?” This invitation for Ethyl to reflect upon her answers helps Ethyl liberate herself from too much certainty about the *truth* of her understanding of Alice.

Ethyl “I'd guess 60 to 70%, maybe less.”

Karl “Would you be surprised if she said 80%?” By responding with a different percentage than the client's estimate the therapist plants doubt, which biases the client towards becoming more curious about and attentive to the other's actual experience. If the client's estimate was very high, say 90%, I might ask “Would you be shocked if it was only 55%?”

#### 2.2.2 Comments Regarding Vignette #2

This series of reflexive questions to the internalized Alice prompted Ethyl to expand her imagination about what might be going on in Alice's experience and probably prepared her for what happened afterwards. When I heard a couple of weeks later that Ethyl had canceled the next appointment because “things were better” I called her to seek permission to use the video for teaching and asked about what happened after our session. Upon returning home, Ethyl took Alice out for a coffee and told her about the IOI. Alice was very curious about her mother's answers when speaking as her (Alice) and was “amazed” at how well her mother understood her. As a result, Alice felt much closer to her mother and the relationship between them improved. And as the relationship between Ethyl and Alice improved, the relationship between the sisters improved as well. Consequently, the family collectively felt there was no need for further therapy. In a subsequent meeting with Ethyl (a couple of years later about a different issue) she reported that the improvements in relation to Alice had been sustained.

### 2.3 Clinical Vignette #3—Interviewing the Distributed Self of a Child^5^

A single mother brought her 5‐year‐old son to a suburban clinic because he had been setting fires for several months. He persisted in the fire‐setting even though some of the fires got out of control to the extent that he had sustained burns that required skin grafting. One of the interesting developments that the mother noted was that the boy stopped talking after the first major fire. When he was pressed to speak at home, the most he would do was sort of giggle. The same giggling occurred when I tried to speak with him during our meeting. I decided to try to interview the boy as an internalized other within the mother while he observed. The mother (Nancy) held the boy (Freddy) on her lap as I spoke with her.

#### 2.3.1 Interview

Karl To Nancy, “Is it OK with you if I try speaking to Freddy within you, to get to know him a bit?”

Nancy “What do you mean?”

Karl “When we interact with someone repeatedly, we gradually form an image of them within ourselves. So, you've developed some impressions of Freddy within yourself including what's going on for him, and that's now part of you. Your internalized Freddy is never going to be exactly the same as what's going on inside him, but there is going to be some overlap between his experience, and your experience of his experience. So, by speaking with the Freddy within you, I might be able to understand him a bit. Are you willing to help me in this way?”

Nancy “Sure. Of course.”

Karl To Freddy within Nancy, “Freddy, how do you go about starting these fires? Do you use matches? A lighter? Or what?” I assumed that Nancy had some knowledge of this, and I wanted to bring forth some concrete behavior to indirectly invite Freddy into some relevant memories.

Nancy Answering as Freddy, “I use matches. My Mom won't let me have a lighter.”

Karl To Freddy within Nancy, “And how do you get the fire going Freddy? Do you use paper, little twigs, or something else?” Freddy, previously distracted, begins looking at me intently.

Nancy Answering as Freddy, “Mostly I use the flyers that the postman drops off in the mail.”

Karl To Freddy within Nancy, “And what happens to you, Freddy, when the fire gets going? Do you get excited or what?”

Nancy Answering as Freddy, “Yah, it's nice and warm, and exciting to watch!” Freddy's eyes open wide, and a slight smile crosses his face.

Karl To Freddy within Nancy, “If the fire gets too big, do you try to put it out? … What do you use to put it out?” Freddy turns his head to look at his mother.

Nancy Answering as Freddy, “Yah, one time I tried to use a blanket, but I got into trouble for ruining the blanket.”

Karl To Freddy within Nancy, “And if the fire really gets out of control, do you get scared?”

Nancy Answering as Freddy, “Yah, I get really, really scared!” At this point Freddy begins sobbing, and Nancy, shocked at his reaction, rocks him back and forth as he folds into her body.

Nancy Speaking spontaneously as herself, “This is amazing! I've never seen him cry like this since that first big fire!”

#### 2.3.2 Comments Regarding Vignette #3

This interview took place in Ireland when I was visiting with The Fifth Province team, and I did not have access to any follow‐up. However, in making sense of what happened, it seems as though Freddy resonated with his DS within his mother—that is, he connected with himself as he existed within her. The details of her knowledge of his actions were sufficient for him to relate to his own behavior. Hearing her give words to his experiences when she was speaking as Freddy probably rendered those experiences more fully available to him. I suspect that when Nancy expressed his emotion of fear, she provided him with the words he needed to move past an emotional block that had previously left him relatively frozen in his capacity to express himself and interact with others. Naming and expressing his fear in this way appeared to set him free and allowed a backlog of sadness to pour out when he began sobbing. In other words, as we get to know how we exist in others, we not only get to know more about ourselves, we can also potentially move forward in our own development as persons.

### 2.4 Clinical Vignette #4—Interviewing the Distributed Self of the Father

Returning briefly to the introductory example of George and Bonnie, I subsequently did meet with them conjointly. As part of the therapy, I also interviewed the father as an internalized other within the daughter. In this conjoint interview, I implicitly invited George to meet some aspects of his distributed self as he existed in Bonnie.

#### 2.4.1 Interview

Karl Interviewing Bonnie but addressing George as her internalized father, “George, what is it about Bonnie that you appreciate the most?” Starting with such appreciative questions supports a positive emotional context which helps create some safety and encourages more openness in George's listening.

Bonnie Answering as George, “I … don't know.” Bonnie's difficulty in answering invites the observing George to possibly recognize that he seldom expresses his appreciation of her.

Karl Continuing to address the internalized George, “I realize that you care about Bonnie a lot, and that you want to protect her, what is it about her going out that worries you the most?” Adolescent children are often far more aware of their parents' worries than most parents realize. Inviting Bonnie to disclose this awareness could have a useful effect on her father.

Bonnie Answering as George, “I'm worried that she's going to have sex and get pregnant.” George was shocked! He had never even mentioned sex to his daughter for fear that doing so might provoke her to become sexually active.

Karl To George within Bonnie, “So George, is that why you won't let her go to her friend's parties?” This question invites an explicit awareness of the connection between his fears and his controlling practices which is useful for both father and daughter to recognize.

Bonnie Answering as George, “Yah, and I think she's going to take drugs like all the other kids.”

Karl To George within Bonnie, “Suppose instead of simply refusing to allow her to go to the party, you asked her about her own concerns for her safety and how she would respond if a guy made a pass at her, or offered her some drugs, how do you think she would respond to you?” This is an embedded suggestion question intended for the observing George.

Bonnie Answering as George, “She'd probably appreciate that a lot!” Again, this was quite a surprise for George, but a huge relief for him as well.

Karl To George within Bonnie, “And what would you appreciate about Bonnie if she was willing to make some changes?” This question focuses more on Bonnie and attempts to bring forth some stabilizing reciprocity in a preferred pattern in the father‐daughter interaction.

Bonnie Answering as George, “It would really be nice if she would let me know where she is going and when she'll be back.” Even more relief came over the father as he recognized that she understood what he needed from her.

#### 2.4.2 Comments Regarding Vignette #4

The therapy went on to co‐construct greater awareness of the limited degree to which the father could influence his daughter's autonomy. This made it possible for him to gradually step back from his controlling efforts, which gave space for her to step forward and take more responsibility for making better choices when she was with her friends. To be sure, other interventions were employed, like externalizing the father's sociocultural assumptions of parental ownership of children (“children belong to their parents”) that contributed to the change process. Nevertheless, the growth of understanding through interviewing George's DS in Bonnie seemed to have been instrumental in enabling a major improvement that occurred in the father‐daughter relationship.

## 3 Interviewing Both the IO and the DS in Couple Work

**FIGURE 2:**
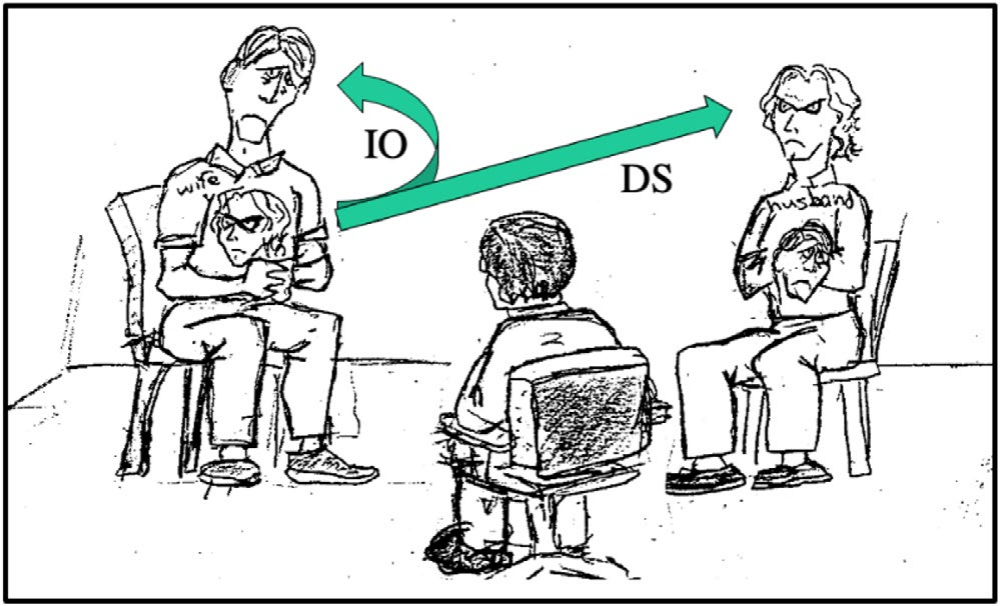
Interviewing both the internalized other and the distributed self.

Over the years, I have gradually developed a sequence of statements and questions that I have found useful for IOI with couples, including an important *debriefing* process. This series of questions could be seen as a scaffold that can be drawn upon in conjoint work, not only with couples, but with any kind of dyad—a parent and child, two siblings, two workmates, a supervisor and supervisee, an employer and employee, etc. The sequence is not intended to be used in a cookbook manner but may serve as a background resource for generating reflexive questions. Every couple's situation is unique, and it is incumbent upon the therapist to respond to that uniqueness to optimize the couple's specific possibilities. At the same time, however, there are similarities across relationships, which makes it feasible to draw from a series of generic questions. In what follows, I offer a possible sequence of reflexive questions alongside common client responses drawn from a composite of IO interviews with several couples. Let us assume I am meeting with Ann and John, a heterosexual couple, as illustrated in Figure 2.^6^

### 3.1 Interview With Ann and John

Karl To the couple, “Would you be open to an experiment about how well you understand each other's experiences?”

Either partner might respond “What do you mean?” Depending on the couple's degree of psychological mindedness and interest, I might launch into a substantial explanation. However, saying less is usually better, since too much explaining sometimes triggers unnecessary anxiety.

Karl Expanding on what I said to Nancy in the earlier transcript I might say, “When we interact with someone in an ongoing relationship, we automatically build up an impression of that person's thoughts, feelings, hopes, intentions, etc. within ourselves. Over time these impressions grow into a complex constellation of the other as *a whole person* within our memory. This *internalized other* is never exactly the same as the outside *actual other* but there will be some overlap or congruence between them. It is sometimes interesting to become aware of which thoughts and feelings of one's internalized other fit with the experiences of the actual other, and which do not. The degree of congruence, or mismatch, in these understandings can be explored by interviewing one person as if he or she were the other, and then later clarifying the similarities and differences between the internalized other and the actual other. Are you willing to give it a try?”

Ann and John Responding, “OK” … “Sure, why not?”

Karl To the couple, “Who would like to go first?” I typically offer the dyad this choice unless there is some good reason to suggest one person should begin. For instance, if there appear to be major issues of sexism influencing the heterosexual relationship it is useful to interview the internalized wife within the husband first to help him get in touch with its problematic effects upon her. Let's assume John volunteers to go first, so I start with him.

Karl To John, “I'd like to begin talking with you as if you were Ann. I'm asking you to answer from your experience of her experience using the *I position* of Ann. Obviously, you don't know exactly what she thinks and feels, but you have some intuitive hunches about what may be going on for her, and I'd like you to respond from those hunches. I am not asking you to role play her outer behavior but to speak from her inner experiences. You might come up with something she has never thought of consciously, but it still might fit for her.”

Karl Turning to Ann, “And while I'm speaking with John's internalized Ann, I'd like you to reflect upon your own responses to my questions so we can discuss the similarities and differences afterwards. Would you like a pen and paper to take some notes along the way? Or do you have a good memory?”

Karl Looking at and speaking to John but addressing Ann within him

“Ann, how did you feel about coming to today's session?” Beginning with this easy question to the internalized Ann‐within‐John about her recent experience helps John ease into speaking from her perspective. In coming to the session together, John probably got a sense about how she felt about the meeting.

John Speaking as Ann, “I was a bit nervous about not being heard and understood.” The observing Ann implicitly meets her DS within John.

Karl To Ann within John, “And how do you feel about how our conversation has been going so far, Ann?”

John Answering as Ann, “It's been OK–better than I expected.”

Karl To Ann within John, “How do you feel about John being willing to engage in this experiment?”

John Answering as Ann, “I'm pleased that he's open to it – especially since he didn't want to come for therapy in the first place.”

Karl To Ann within John, “Ann, what is it about John that you respect and admire the most?” This question invites John to consider positive aspects of his reputation as they exist within Ann – he begins exploring what she values in his distributed‐self‐within‐Ann. His answer will reflect both how well she communicates with him about himself and how well he listens to her. Partners often have difficulty responding to such questions because clients typically come to therapy immersed in problem saturated stories – nonetheless these questions usually provoke generative reflections for both parties.

John Struggling to answer as Ann, “Ahh … he can be gentle and kind when he wants to.”

Karl To Ann within John, “What are his special talents and gifts? … What do you like about him as a person? … What was it about John that drew you to him in the first place, Ann?” As noted in the previous example with Bonnie, these questions and their affirming answers privilege positive emotions and create a bit of safety in this unusual kind of conversation.

John Answering as Ann, “Oh, I don't know. … He's trustworthy and honest.” The observing Ann encounters her DS making value judgments about him and his behavior, which are often unspoken, yet often very meaningful.

After a few questions along this theme, I typically shift to explore the relationship between John and Ann, moving slowly from what is currently valued to what is of concern.

Karl To Ann within John, “Ann, what is it that you already have in your relationship with John that you value and want to preserve, and perhaps even strengthen? … What else?”

John Answering as Ann, “I think we have a strong sense of loyalty. We have each other's back.”

Karl To Ann within John, “Ann, what is it in your relationship with John that you are the most worried about? … What else are you concerned about?” Again, Ann meets her DS.

John Answering as Ann, “I'm worried that we're drifting apart. … We seem to have less in common than we used to.” Optionally, one could extend this exploration by enquiring about the perspectives of others who know the couple, especially if they are present in the interview. (“Ann, how do you think your kids are being affected by your relationship with John? … Ann, what do your friends appreciate, or have concerns, about your relationship with John?”). Still addressing John's internalized Ann, I might then shift to enquire about her needs, desires, and vulnerabilities drawing on issues talked about prior to the IOI.

Karl To Ann within John, “Ann, what is it that you need the most, at this time in your life? … What unfulfilled desires do you struggle with, Ann? … What kinds of things seem to upset you the most?” These questions invite John to empathize more deeply with Ann's inner experiences.

John Answering as Ann, “I need to feel more connected. … I feel very lonely at times.” Once again, Ann implicitly meets herself as she exists in John.

Karl To Ann within John, “Ann, suppose John were willing and able to make some changes in the way he relates to you, what single change would you appreciate the most?” As noted earlier this is an extremely useful question since it could help John reflect upon what he could do that might make a real difference for Ann. In effect, he gives himself some good advice.

John Answering as Ann, “He could be more patient instead of getting so frustrated with me.”

Karl To Ann within John, “What exactly would John be doing differently if he were to take this change seriously, Ann?… How would you know that he was working on it? … What initiatives would be most convincing for you, Ann?” These follow‐through questions help John operationalize some potential changes in concrete action that he might consider.

John: Answering as Ann, “He could stop raising his voice and avoid yelling at me.”

Karl To Ann within John, “What's it like for you inside, Ann, when he yells at you?”

John Answering as Ann, “I get quite anxious and scared when he yells. It reminds me of my father who was quite violent at times.” The hope is that the more deeply John gets in touch with her negative experience of his behavior, the more likely he might learn to avoid managing his frustration and anger in that manner.

Karl To Ann within John, “What would you like John to do instead?”

John Answering as Ann, “I think he should give himself a time out to calm down and talk with me about it later.”

Karl To Ann within John, “Let's say John was willing and able to make further changes, what other changes would you appreciate, Ann?”

John Answering as Ann, “He could suggest things we could do together – like going out to movies or concerts.” These preferences for change could be explored further in detail depending on what emerges.

Karl To Ann within John, “What else could I ask you about, Ann, that might help me understand your relationship with John more fully?” This is a meta question (a question about questions) which invites John to bring up any relevant issues he might like explored. If something significant does come up, I follow the lead with further reflexive questions. Otherwise, I move to closure and debriefing.

Karl Ending the IOI of Ann within John, “Thank you for your thoughtful responses, Ann. … Goodbye for now. …. And hello to John! … Let me talk with you again as yourself. What was it like for you to speak from Ann's experience? … Was it more difficult than you expected, or easier than you thought?”

John Speaking as himself, “It was actually easier than I thought it would be.”

Karl To John, “If you were to estimate, on a scale of 0 to 10, with 0 being unable to get into her experience at all, to 10 being fully into her experience, how far do you feel you were able to go?”

John “Probably a 7.”

Karl “What sense do you have of what may have emerged if you allowed yourself to go deeper into her inner experience?” This leaves John with the realization that more learnings could be harvested from this process if he wanted to explore things further. I have had people ask me to repeat the IOI process to help them enter their partner's experiences more fully. They have wanted to understand better but were unable to formulate the relevant questions for themselves.

John “Maybe I would have gotten a better sense of how afraid she actually is of my anger.”

Karl “What percentage of the answers that you gave, speaking as Ann, do you imagine resonated with her, and about which she was probably thinking ‘Yeah, that's about how I feel’? What's your honest guess?”

John “I'd say 65%” If significant others (like older children) were observing, I might also ask them what percentage they imagine might fit for Ann before asking Ann herself.

Karl To the actual Ann, “And Ann, roughly what proportion of his answers speaking as you, fit for you as you were listening? … What percentage came to mind, before John (and the kids) gave his (their) guess?” Estimates of fit or accuracy describe a different dimension than depth of understanding—both reflections yield interesting information for all participants.

Ann “I'd say 85%”

Karl To Ann, “Wow, that's pretty good! … In the 85% that fit for you, was there anything that *surprised* you about what he understood about your experience?” There may or may not be surprises, but if there are, it is good for John to hear what they were.

Ann “Yes, I didn't realize that he knew I sometimes feel quite lonely. I try to hide it from him.” If there were no surprises, then I move on to ask Ann about the answers that did fit.

Karl To Ann, “What among the 85% that fit, when he was speaking as you, were you *most pleased* to hear that he seemed to understand?”

Ann “I'm glad he realizes that I get quite scared when he gets angry. It really triggers me.” I enquire further about what else fit that was meaningful for Ann before moving on.

Karl Then turning briefly to John, “Are you interested in hearing which answers didn't fit for her?” In my experience the interviewee almost always wants to hear. (If he answers “No” I would leave things there and suggest they might discuss the IOI further on their own.)

John “Yes, I'd really like to know!”

Karl Back to Ann, “What were the main things that didn't fit for you? … How would you have responded to those questions differently?”

Ann “Oh, I wasn't nervous about coming to the session today at all! I feel heard here. … And I don't feel like we're drifting apart. … I just feel I need to take space for myself, especially when he gets frustrated with me.”

Karl To John, “What do you understand as the difference between Ann's actual experience here and your experience of her experience?”

John “I guess she's not distancing from me so much as she's protecting herself.”

Ann “Yes, that's it.”

Karl To John, “Were there any questions I asked you as Ann that you did your best to answer but that she hasn't commented on, about which you would be interested in hearing her response? … What are you most curious about?”

John “Oh yah, those questions at the beginning about what you appreciate about me.”

Ann “Oh, I guess I don't tell you often enough how much I appreciate you. … How hard you work to support us. … You're incredibly reliable as a provider. … Most of the time you're extremely kind and generous. And sometimes you're even fun!”

John “Oh, thank you! That's good to hear.”

### 3.2 Comments on the Couple IOI

If time permits, I then interview John as an internalized other within Ann, using a similar sequence. Alternatively, this could be done in another session, or be left out altogether. The aim is to allow the process to unfold slowly and give the IO lots of time to reflect. A therapist should never feel compelled to follow this full sequence of questions—I never manage to do so myself. The sequence is simply a template from which one can draw useful reflexive questions. Use your intuition to guide your choice of question—your tacit knowledge is always larger than your conscious knowledge anyway. IOI questions often require deep thought and intense reflection by the interviewee. If a person appears to be struggling to engage with the experience of the IO, I often reassure them: “Take your time … these are difficult questions.” Sometimes I'll suggest the person close their eyes and look deeply into their experience of the IO's experience before responding.

An interesting complication sometimes arises during the IOI process when there is a significant power differential between the members of a dyad. For instance, if John happened to be a very domineering male, he might answer in Ann's voice the way *he would like her to think and feel*. Such responses are seldom conscious but nonetheless undermine the usefulness of the experiment. At such a point, a good question to ask to the internalized Ann might be: “Ann, suppose you got the impression that John was answering these questions the way he wants you to think and feel, rather than how you actually feel; how would that make you feel, Ann?” This question usually triggers sufficient awareness for John to abandon his implicit efforts to manage her experience, and the interview typically moves forward as intended. There is rarely any need to stop the process or to confront John (although a similar question may be needed again if the same complication re‐emerges).

At the end of an IOI session, it is best to avoid asking if the process was helpful, since such questions tend to intellectualize the experience which is usually quite emotional for both parties. Moreover, such an evaluation is often more for the benefit of the therapist than for the client. It is usually better to simply allow clients to sit with, soak up, and integrate their experience of the rich process. Interestingly, in the next interview clients sometimes mention that “things are better” but they don't know why. My hunch is that by living slightly more in the experience of the other they spontaneously respond to one another in preferred ways more often.

## 4 A Few Words of Caution

Given the uncommon nature of IOI, clients are more vulnerable than usual during such an interview. When speaking in the voice of the other, their usual defenses are not as readily available to them when asked an intrusive or unwelcome question. For instance, I have encountered situations where clients have disclosed secrets in the voice of an IO that they would not have disclosed in their own voice. Because of this vulnerability, it is extremely important that the therapist adopt a strong ethical stance of genuine respect and caring for the well‐being of the client *before* embarking on such an experiment. In my opinion, if a therapist is not able to feel warmly towards the client (perhaps because the client has engaged in some horrific violence or abuse) they should not use this method—they cannot trust themselves to avoid asking questions that could have demeaning or counter‐therapeutic effects upon the client.

IOI is also contraindicated with clients who are compromised in their capacity for reality testing. During psychotic episodes, one's boundaries and identity may be unclear or may be confused. Clients may not be able to clearly distinguish self from other. This is not to say that this method cannot be used effectively with clients who have previously suffered with psychotic episodes. I have used IOI to benefit clients who have been diagnosed with schizophreniform episodes, but only when they were well integrated again. One should always be prepared to abandon the IOI process at any time when one intuits that the process is becoming counter‐therapeutic and resume one's more usual patterns of therapeutic interviewing.

### 4.1 A Few Comments About Theory

Becoming the *other*, or interviewing an IO, is a practice which anyone can engage in without any theoretical preparation. Indeed, it is remarkable to see how children in spontaneous play, as young as 2 or 3 years, can step into the identity of a parent and address a doll as if the doll were the self. In other words, the phenomenon of internalizing others appears to occur as a pancultural developmental process. However, as we grow into adulthood, our sense of becoming a *separate self* tends to obscure this process, especially in cultures that have drifted toward prioritizing individuality. As a result, we often lose sight of the interpersonal understandings that we have internalized over the years. Subscribing to some relevant theories could help counter this individualistic obfuscation and support one's confidence in initiating an IOI process.

What helps me in this kind of interviewing is a perspective of the human mind being first and foremost a social phenomenon, and secondarily psychological—a perspective endorsed by Gregory Bateson (1972) and Maturana and Varela (1979, 1987). In his theory of knowledge, Maturana claims that we humans, as complex cognizing biological systems, *bring forth* our realities through conversation. Even though this conversation is enacted by biological beings, it is through language in the social domain that we draw distinctions, become observers, generate explanations, and eventually exercise choice. Some colleagues and I at the Calgary Family Therapy Centre have come to describe our systemic work as Bringforthist Therapy (Tomm et al. in press) which is informed by both social constructionism (Gergen 1999) and bringforthism (Maturana and Varela 1979, 1987). Bringforthism, as a paradigm of knowledge, is consistent with social constructionism but is grounded in *both* biology and sociology. While the distinctions we draw arise in the social domain, they depend on our bodies to be enacted.

Allow me to offer one more clinical example to illustrate how we bring forth embodied realities in conversation. Several years ago, a therapist invited me to interview a woman in her 30s who had struggled with bulimia since her teens and had difficulties maintaining relationships with men. She had “disgraced” her family of origin by getting pregnant as a teenager and was cloistered in secrecy until she delivered the child, who was immediately given up for adoption. She tried to suppress all memories of that period in her life, yet she continued to live with strong feelings of shame and guilt. Given that it was often her mother who passed judgment upon her for having “sinned,” I briefly interviewed her internalized mother to try to deconstruct the pattern of internalized judgment. While doing so, it occurred to me that if I interviewed the child that she had given life to, she might encounter a more substantive basis to feel positively about her earlier experience. When I raised the possibility of interviewing her internalized child, she recoiled and declined the invitation. I asked if it would be OK if I interviewed the child as an IO within her therapist. She became curious and supported that option. However, I needed a name to speak to the internalized child. The client had not given the child a name at birth and even claimed not to know if it was a girl or boy. We eventually settled on the name Sam, which could represent either Samantha or Samuel. I then proceeded to interview Sam as an IO within the therapist while the client observed.

#### 4.1.1 Interview

Karl Addressing Sam within the therapist, “Sam, how do you feel about your birth mother having given you life and bringing you into this world?”

Therapist Speaking as Sam, “I am grateful to her. I wouldn't exist if it wasn't for her!”

Karl To Sam within the therapist, “Sam, what do you think about all the judgements that were heaped upon your bio‐mom as she bore you?”

Therapist As Sam, “It was so unfair. And it is so sad when family members cannot be more accepting of what happens to us in life.”

Karl To Sam, “What would you like to say to your bio‐mom if you had the opportunity to do so Sam?”

Therapist As Sam, “Oh, Thank you! Thank you so much! I could never thank you enough! You have given me life, and that is the greatest gift of all!”

The client appeared to become enlivened as she watched this. I continued speaking in this manner with the therapist for a few minutes and then turned to ask the client if she was now ready for me to interview Sam within her as well, and she said “Yes.” So, I proceeded to ask a similar set of questions to the client's internalized Sam. This proved to be a deeply moving experience for her. By the end of the session, she was almost vibrating with excitement. I happened to meet her therapist again a couple of years later and she reported how this IOI had become a turning point in the client's life; the bulimia subsided, and for the first time, she was able to establish a stable relationship with a male partner.

When Heinz von Foerster, a well‐known radical constructivist, heard me recount this interview, he insisted that I had brought Sam into existence through my questions—that I had actually “constructed” Sam. I regard Heinz's theoretical perspective as compelling but a bit too extreme. The client's embodied sensorimotor experiences in having borne the child afforded me a coherent basis to co‐construct Sam together with her. The biology needed to first be there as a substratum to enable a viable and enduring social construction to be brought forth.

### 4.2 A Few Words of Encouragement

There are many, many ways that this method of interviewing may be implemented in therapy. I have interviewed several internalized others in the same person, the same internalized other in multiple family members, a parent within a child as young as 4, a deceased person as an internalized other in a bereaved person, a diagnosis as an internalized other, and even an internalized other of an internalized other. The possibilities are endless, and undoubtedly other therapeutic applications have been and will be explored in the future.

For those of you out there who have never employed this kind of interviewing, you may find yourself on the cusp of a not‐yet‐realized potential as an interviewer. When I present this kind of interviewing in workshops, I encourage participants to engage in an IOI exercise with one another as a learning experience. The interviewee is entitled to decide which IO they would like to have interviewed. If you wanted an affirming professional experience, you might invite the interviewer to speak with a client who has had a good therapeutic outcome in working with you. The interviewer could ask your internalized client what you did in the therapy that may have enabled that outcome. If you wanted a more intense personal experience of what it might be like to be such a client, the interviewer could be invited to interview a close family member with whom you have some issues. During such an exercise, the interviewer is not expected to be proficient in formulating excellent questions but to practice developing their skills. They are entitled to make “mistakes” which could contribute to learnings for all participants.

Given that this exercise option may not be immediately available to you as the reader, you could consider taking a moment now to formulate a few questions to any IO within yourself with whom you might wish to explore some relational understandings in your imagination. Who might you be a bit curious about? Perhaps you could begin by asking them: “My dear … (name of your IO) what is it about me that you appreciate? …” At the very least, a few honest reflections upon such questions will nudge you towards a more relational way of being. Go ahead, give it a quick try!

## Conflicts of Interest

The author declares no conflicts of interest.

## Endnotes

^1^
All the given names used in this paper are pseudonyms to protect the privacy of the clients.

^2^
I never did take up Gestalt work, partly because I distanced myself from Fritz after he frightened my then one‐year‐old daughter when he persisted in trying to lift her up while she was spooked by his huge beard and was struggling to escape his grasp.

^3^
An extremely abbreviated version of this interview was first published in the Common Language for Psychotherapy (cpl) Procedures: www.commonlanguagepsychotherapy.org. The dialogue reported in this interview was reconstructed from memory.

^4^
Parts of this video were presented at a Conference in New York in 1996 and stimulated an interesting written exchange with Salvador Minuchin about the connections between family therapy and narrative therapy in the *Journal of Marital and Family Therapy* (Minuchin 1998; Tomm 1998).

^5^
While the method of addressing the IO is the same, the title for this vignette indicates that the focus for intended therapeutic change here is mainly on the observing other, rather than the interviewee.

^6^
My nephew, Matthew Tomm, drew this cartoon (without the arrows) when he was 12 years old, capturing the concepts very nicely.
